# Shionone-Targeted Pneumolysin to Ameliorate Acute Lung Injury Induced by *Streptococcus pneumoniae* In Vivo and In Vitro

**DOI:** 10.3390/molecules27196258

**Published:** 2022-09-23

**Authors:** Runbao Du, Tian Wang, Hongfa Lv, Yinuo Zou, Xiaoning Hou, Nana Hou, Peng Zhang, Hongen Li, Gefu Chi

**Affiliations:** 1 The Affiliated Hospital of Inner Mongolia Medical University, Hohhot 010107, China; 2 Department of Thoracic Surgery, The First Hospital of Jilin University, Changchun 130062, China; 3 State Key Laboratory for Zoonotic Diseases, Key Laboratory for Zoonosis Research, College of Veterinary Medicine, Jilin University, Changchun 130062, China; 4 Department of Laboratory Animals, College of Animal Sciences, Jilin University, Changchun 130062, China; 5 Department of Ophthalmology, The First Medical Center, Chinese People’s Liberation Army General Hospital, Beijing 100039, China

**Keywords:** shionone, *Streptococcus pneumoniae*, pneumolysin, oligomerization, antivirulence

## Abstract

*Streptococcus pneumoniae* (*S. pneumoniae*), as a Gram-positive bacterium, can cause severe bacterial pneumonia, and result in high morbidity and mortality in infected people. Meanwhile, isolated drug-resistant *S. pneumoniae* is growing, which raises concerns about strategies for combatting *S. pneumoniae* infection. To disturb *S. pneumoniae* pathogenicity and its drug-resistance, developing novel anti-infective strategies or compounds is urgent. In this study, the anti-infective effect of shionone was explored. A minimum inhibitory concentration (MIC) assay and growth curve determination were performed to evaluate the effect of the tetracyclic triterpenoid compound shionone against *S. pneumoniae.* Hemolysis tests, western blotting, oligomerization inhibition assays, and molecular docking were carried out to explore the anti-infective mechanism of shionone. Moreover, the protective effect of shionone was also confirmed in a mousepneumonia model. The results showed that the excellent hemolytic inhibitory activity of shionone was observed at less than 8 μg/mL. Meanwhile, shionone could disturb the oligomerization of pneumolysin (PLY) but did not interfere with PLY expression at less than 4 μg/mL. Molecular docking suggested that shionone targeted the ASP-59, ILE-60, THR-57, PHE-344, and ASN-346 amino acid sites to reduce *S. pneumoniae* pathogenicity. Furthermore, shionone alleviated lung histopathologic injury and decreased lung bacterial colonization in vivo. The above results showed that shionone could bind to the PLY active pocket under the concentrations of 8 μg/mL and neutralize PLY hemolysis activity to reduce *S. pneumoniae* pathogenicity in vitro and in vivo.

## 1. Introduction

*S. pneumoniae*, as a Gram-positive opportunistic pathogen, often colonizes animals or the human nasopharynx and can cause bacterial pneumonia to spread rapidly worldwide [1,2,3]. Older adults, children, or immunocompromised people are prone to infection by *S. pneumoniae*. Depending on the infection site of *S. pneumoniae*, it could trigger pneumonia, otitis media, septicemia, meningitis, or other diseases. According to emerging research, once COVID-19 prevention efforts were loosened, the incidence of invasive pneumococcal disease began to rise once more [4]. Currently, penicillin is used to treat invasive pneumococcal disease in clinical practice. However, the emergence of bacterial resistance and imbalance of intestinal flora limit the clinical application of antibiotics [5]. Therefore, the development of new drugs or novel strategies to combat *S. pneumoniae* infection deserves national attention and action.

The pathogenicity of *S. pneumoniae* depends mainly on virulence factors including polysaccharide pods, adhesion factors, invasion factors, or transport proteins [6]. Pneumolysin (PLY), a 53 kDa cholesterol-dependent cytolytic hemolysin, is an important virulence factor of *S. pneumoniae* that can bind to cholesterol on the membrane and then oligomerize into *β*-barrel-shaped transmembrane pores, causing target cell lysis and death [7]. Many studies have revealed that natural small molecule compounds, such as acacetin and verbascoside, targeting PLY could alleviate pneumococcal pneumonia injury [8,9] and that PLY could be an ideal target for screening inhibitors to defend against *S. pneumoniae* infection.

Shionone is a tetracyclic triterpenoid compound found in the *aster tataricus*, a plant of the Asteraceae family, and has medicinal value in treating wind chills, coughs, and asthma [10]. It is white crystal or dust and dissolved easily in petroleum ether, chloroform, and other organic solvents. Shionone could interfere with the TNF, IL-17, and Toll-like receptor signaling pathways by inhibiting CXCR4, ICAM1, and other highly expressed genes to fight against COVID-19 in vivo [11]. Meanwhile, shionone has been reported to attenuate NLRP3 inflammatory vesicle-mediated cell scorching [12] and inhibit human breast cancer cell growth, migration, and invasion by inducing apoptosis and inhibiting the induction of the MEK/ERK and Stat 3 signaling pathways [13]. However, there are no reports on the effect of shionone against *S. pneumoniae* infection.

In this study, the elementary mechanism of shionone against *S. pneumoniae* infection was explored firstly mainly via hemolysis tests, oligomerization inhibition assays, and molecular docking in vitro and establishing acute lung injury (ALI) in mice models to probe the protective effect of shionone in vivo. This study lays the foundations for the clinical application of shionone and provides a novel strategy to fight *S. pneumoniae* infection.

## 2. Results

### 2.1. Shionone Inhibits PLY Hemolytic Activity

**Figure 1:**
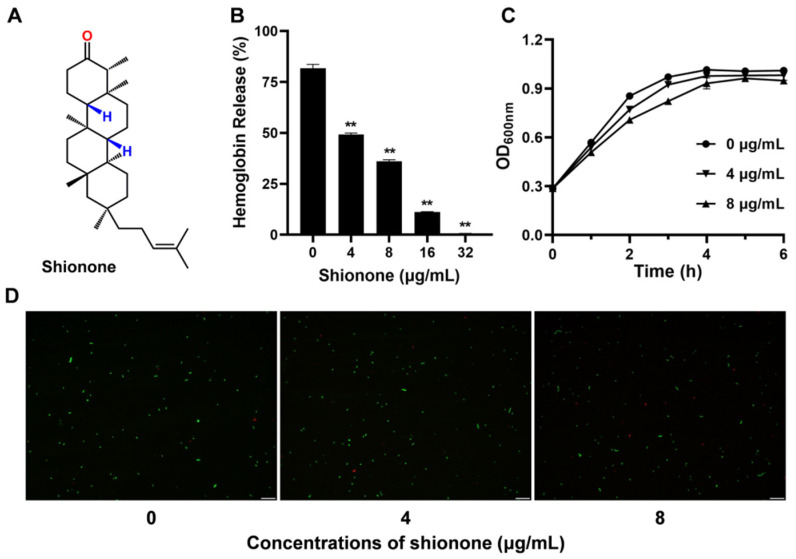
Shionone inhibits PLY hemolytic activity and does not affect *S. pneumoniae* growth at less than 8 μg/mL. (**A**) Shionone chemical structure formula. (**B**) Shionone exhibits a concentration-dependent inhibitory effect on the hemolytic activity of PLY. (**C**) The growth curves of *S. pneumoniae* were determined under shionone treatment with different concentrations (0 μg/mL, 4 μg/mL and 8 μg/mL). (**D**) The fluorescence images of *S. pneumoniae* treated with different concentrations of shionone (0 μg/mL, 4 μg/mL, and 8 μg/mL) for 6 h. Green in the image represents live cells, and red represents dead cells. The significance level was expressed as follows: ** *p* < 0.01.

Shionone is a tetracyclic triterpenoid with a unique six-membered tetracyclic skeleton and a 3-oxo-4-monomethyl structure, as shown in Figure 1A. The results of the hemolysis test showed that shionone significantly inhibited purified PLY hemolytic activity at 4 μg/mL (Figure 1B). Surprisingly, PLY hemolytic activity was completely inhibited by shionone at 32 μg/mL (Figure 1B). The MIC value of shionone against *S. pneumoniae* was 128 μg/mL. According to the results of the growth curve in Figure 1C, shionone had no effect on the growth of *S. pneumoniae* under the concentrations of 8 μg/mL. To visualize the effect of shionone against *S. pneumoniae* growth, BacLight LIVE/DEAD analysis was performed. Consistent with the results of the growth curve assay, shionone did not affect *S. pneumoniae* growth at less than 8 μg/mL (Figure 1D). The amount of living bacteria (dyed green) treated with 4 μg/mL or 8 μg/mL shionone was in line with the blank control (Figure 1D). In summary, shionone inhibited PLY hemolytic activity but had no effect on the growth of *S. pneumoniae* at less than 8 μg/mL.

### 2.2. Shionone Alleviates A549 Cell Injury Induced by PLY

**Figure 2:**
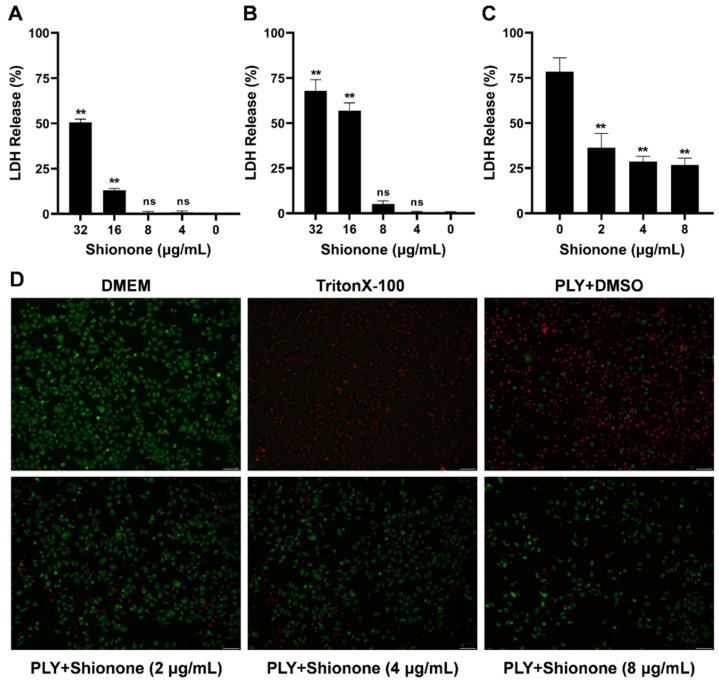
Shionone has no cytotoxicity on mammalian cells and alleviates PLY-mediated cell injury at less than 8 μg/mL. (**A**,**B**) Cytotoxicity of different concentrations of shionone (4–32 μg/mL) in the A549 cell lines and J774 cell lines. (**C**) LDH release in A549 cells induced by PLY with or without shionone treatment. (**D**) Visualization of the protective effect of shionone on A549 cells stained with the LIVE/DEADTM Viability/Cytotoxicity Kit (green for live cells, red for dead cells). The significance level was expressed as follows: ** *p* < 0.01; NS, no significant difference.

To further explore whether shionone could alleviate the cell injury induced by PLY, cytotoxicity and live/dead assays were performed. As shown in Figure 2A,B, shionone had no cytotoxicity to A549 and J774 cells at less than 8 μg/mL. Moreover, PLY more easily attached to red blood cells or lung epithelial cell membrane surfaces, causing cell lysis and death. Therefore, A549 cells were selected to explore the protective effect of shionone on cell injury induced by PLY. As shown in Figure 2C, 0.48 μM PLY caused 75% A549 cell injury, and shionone neutralized PLY activity to obviously alleviate cell injury at less than 8 μg/mL. As shown in Figure 2D, the cytotoxic effect of PLY on A549 cells was similar to that of Triton X-100, which caused more cell injury (dyed red) compared to the blank control with DMEM treatment. Interestingly, shionone clearly alleviated cell injury induced by PLY at less than 8 μg/mL, as shown in Figure 2D, because many living cells were dyed green. These results suggested that shionone could alleviate A549 cell injury by neutralizing PLY activity.

### 2.3. Shionone Neutralizes Toxicity by Inhibiting PLY Oligomerization

**Figure 3:**
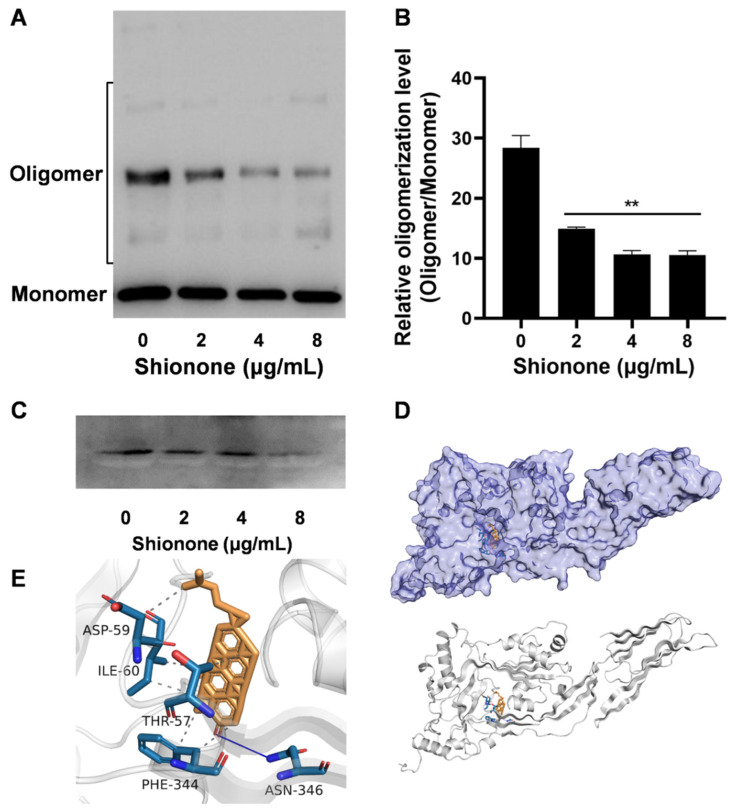
Shionone inhibits virulence of *S. pneumoniae* by targeting the oligomerization of PLY. (**A**) The oligomerization of PLY was detected by western blotting treated with or without shionone. (**B**) Densitometric analysis of PLY oligomerization with different concentrations of shionone treatment. (**C**) The expression level of PLY determined after *S. pneumoniae* was cocultured with different concentrations of shionone for 6 h. (**D**,**E**) Shionone interacts with the active pocket of PLY and targets amino acid sites ASP-59, ILE-60, THR-57, PHE-344, and ASN-346. The significance level was expressed as follows: ** *p* < 0.01.

PLY can punch holes in tissue cells to help *S. pneumoniae* enter the interior of the respiratory tract and cause damage to lung tissue [14]. One of the key steps in pneumolysin’s virulent action is oligomerization [15]. Therefore, we examined whether shionone exerts an antitoxic effect by blocking PLY oligomerization. As shown in Figure 3A, oligomerization gradually decreased as the concentration of shionone increased. This is supported by the optical density analysis results in Figure 3B. In addition, western blot results showed that shionone hardly inhibited the expression of PLY at less than 4 μg/mL, as shown in Figure 3C. Molecular docking was performed to search the binding site of shionone on PLY. The binding affinities (ΔG (kcal/mol)) and inhibitor constant (Ki (nM)) were −12.9 kcal/moL and 0.342 nM, respectively (Supplementary Table S1). The overview surface modeling between PLY and shionone was shown in Figure 3D, upper panel. In order to show the combination clearly, a protein skeleton model was shown in Figure 3D, bottom panel. The molecular docking results also showed that the potential targets of shionone on PLY were ASP-59, ILE-60, THR-57, PHE-344, and ASN-346 (Figure 3E). ASP-59, ILE-60, THR-57, and PHE-344 were involved in hydrophobic interactions, whereas ASN-346 was only involved in hydrogen bonds, as shown in Figure 3D,E.

### 2.4. Shionone Alleviates S. pneumoniae Virulence in Mice

**Figure 4:**
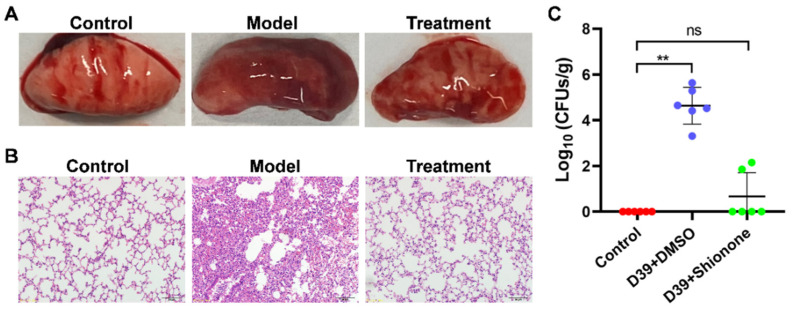
Shionone alleviates mice pneumonia injury induced by *S. pneumoniae*. (**A**,**B**) Gross lung tissue lesions and hematoxylin/eosin staining analysis after infected for 48 h with or without 50 mg/kg shionone treatment. (**C**) Lung colony count of the control, model, and treatment groups after infection for 48 h. The significance level was expressed as follows: ** *p* < 0.01; NS, no significant difference.

A pneumonia model induced by *S. pneumoniae* was established to explore the protective effect of shionone in vivo. After infection for 48 h, significant lung tissue damage with a deep red color and congestion was observed in the model group compared to the control group (Figure 4A). Lung injury was ameliorated with 50 mg/kg shionone treatment, as shown in Figure 4A. The histopathological analysis of the model group demonstrated impaired alveolar structure and inflammatory cell infiltration compared with the control, as shown in Figure 4B. In contrast, alveolar tissue in the treatment group was structurally intact, and associated inflammatory cell infiltration was reduced. Additionally, the results of the lung colony counting assay showed that shionone decreased *S. pneumoniae* lung colonies by approximately 4 × log_10_ units compared with the model group, as shown in Figure 4C. In summary, shionone significantly attenuated the pathological damage caused by *S. pneumoniae* and the associated inflammatory response in mice.

## 3. Discussion

*S. pneumoniae*, an opportunistic pathogen, is more prevalent in children under five years of age and in the elderly [16]. From 2005–2015, 55.8% of mortality in lower respiratory tract infections was attributed to *S. pneumoniae* infection [17]. The effective prevention measures today are the *S. pneumoniae* vaccine, but some studies have shown that these vaccines are not effective against *S. pneumoniae* [18]. Therefore, finding an antibody or vaccine alternative to treat *S. pneumoniae* infection is essential.

PLY, one of the important virulence factors of *S. pneumoniae*, is able to hemolyze cells through oligomerization that is essential for PLY to bind the cholesterol membrane surface [19]. In our study, we first investigated the inhibitory effect of shionone against PLY via a hemolysis assay. When the concentration of shionone reached 32 μg/mL, it almost completely inhibited PLY hemolytic activity (Figure 1B). Moreover, shionone did not inhibit the growth of *S. pneumoniae* at less than 8 μg/mL (Figure 1C). Consistent with a previous study [8], shionone also neutralized PLY activity by disturbing oligimerization formation at less than 8 µg/mL. In addition, 8 µg/mL shionone could reduce the PLY expression level slightly in Figure 3C. These results indicated that shionone not only neutralizes the PLY activity, but also reduces the PLY expression. Lux S/AI-2 quorum sensing system was involved in the biofilm formation, colonization, capsular polysaccharide expression, and *ply* expression [20]. This finding indicated that shionone may interfere with Lux S/AI-2 quorum sensing system to reduce the PLY expression. Cytotoxicity of shionone was observed in cancerous cell line A549 and the J774 cell at over 16 μg/mL, which might be related to the role of the antitumour effect in triterpene compounds [21]. Interestingly, it has no cytotoxicity to mammalian A549 and J774 cell lines at less than 8 μg/mL. This result was consistent with the effect of shionone against RAW264.7 cell lines [22,23]. Meanwhile, shionone (2, 4, 8 μg/mL) was able to protect A549 cells from PLY-mediated cytotoxicity (Figure 2C). Shionone also reduced the pathogenicity of *S. pneumoniae* in vivo (Figure 4C). However, shionone is diffcult to absorb into bloodstream after gavage administration in rats and the peak concentration (C_max_) was only 0.4 μg/mL [24]. After absorption into the blood, it was mainly distributed in the bowel, stomach, and lung tissue without accumulation. Therefore, developing new formulations or changing the route of administration might promote its utilization. In this study, we found that shionone could bind to amino acid sites ASP-59, ILE-60, THR-57, PHE-344, and ASN-346 to disturb the formation of PLY oligomerization and simultaneously alleviate mouse pneumonia injury in vivo. These findings broaden the pharmacological activity of shionone and lay the foundation for new drug developments against *S. pneumoniae*.

## 4. Materials and Methods

### 4.1. Bacterial Strain, Cell Line, and Reagents

The *S. pneumoniae* strain D39 (ATCC49619) used in this study was purchased from American Type Culture Collection (ATCC, Manassas, VA, USA). and cultured according to previous research [8]. Briefly, *S. pneumoniae* was cultured in THY (Todd Hewitt Broth medium containing 2% yeast extract) at 37 °C statistically. Human-derived A549 alveolar adenocarcinoma basal epithelial cells and murine peritoneal macrophage J774 cells were all purchased from American Type Culture Collection (ATCC, Manassas, VA, USA). A549 and J774 cells were cultured based on a previous method [19]. Briefly, both A549 and J774 cells were cultured in Dulbecco’s modified Eagle’s medium/high glucose (DMEM; HyClone, Logan, UT, USA) containing 10% fetal bovine serum and 1% penicillin–streptomycin (MRC, Madrid, Spain) at 37 °C and 5% CO_2_. Dimethyl sulfoxide (DMSO) was purchased from Sigma-Aldrich (St. Louis, MO, USA). Shionone (purity > 98%) was purchased from Shanghai Yuanye Bio-Technology Co., Ltd. (Shanghai, China) an dissolved in DMSO.

### 4.2. Hemolysis Test

Ten microliters of purified pneumolysin (PLY) (0.4 μM) was mixed with different concentrations of shionone (0, 4, 8, 16 and 32 μg/mL) in 965 μL of phosphate-buffered saline (PBS) and then incubated for 60 min at 37 °C. After adding 25 μL of rabbit erythrocytes, the sample was gently mixed and incubated at 37 °C for 10 min. The supernatant was collected at 10,000× *g* for 1 min, and the release of hemoglobin was determined at an optical density of 570 nm (OD_570 nm_) using a microplate reader (Tecan, Melbourne, Austria). A deionized water addition was used as a 100% hemolysis control.

### 4.3. Minimum Inhibitory Concentration (MIC) Determination

The MIC of shionone against *S. pneumoniae* was investigated using a twofold serial dilution method according to the Clinical and Laboratory Standards Institute guidelines [25]. Shionone was serially diluted twofold in THY and mixed with bacterial suspension (5 × 10^5^ CFU/mL) in a sterilized 96-well polypropylene microtiter plate. Then, the plate was placed in an incubator with 5% CO_2_ at 37 °C. The MIC value was defined as the lowest concentration of the compound without visible bacterial growth within 18 to 24 h of incubation at 37 °C.

### 4.4. Growth Curve Assay

The overnight culture of *S. pneumoniae* was diluted (1:100) into fresh THY medium and then cultured at 37 °C statistically until the absorbance values at 600 nm (OD_600 nm_) of the bacterial solution reached 0.3. Shionone was added to the cultures at different concentrations (0, 4 and 8 μg/mL). Meanwhile, an equal volume of DMSO was added as a control. The OD_600 nm_ values of each treatment group were measured every hour.

### 4.5. BacLight LIVE/DEAD Analysis

*S. pneumoniae* was treated with different concentrations of shionone (0, 4, and 8 μg/mL) for 6 h, and then 500 μL of the bacterial suspension was collected at 10,000× *g* for 2 min. After washing with sterile PBS 3 times, the pellet was suspended in 500 μL PBS. According to the instructions of the LIVE/DEAD^TM^ BacLight^TM^ Bacterial Viability Kit (Invitrogen, Waltham, MA, USA), the samples were mixed with 1.5 μL working regent and then incubated in the dark for 15 min. Then, 5 μL bacterial suspension samples were dropped on a slide for imaging with a fluorescence microscope (Fv1000, Olympus).

### 4.6. Cytotoxicity and Live/Dead Assays

A549 cells and J774 cells were inoculated into 96-well cell culture plates (2 × 10^4^ cells/well) and cultured for 12 h. Then, the cells were cultured with different concentrations of shionone (4, 8, 16, and 32 μg/mL) at 37 °C for 5 h. DMEM was used as a negative control, and 0.2% Triton X-100 was used as a positive control. After collecting the cell culture supernatant at 1000× *g* for 10 min, lactic dehydrogenase (LDH) detection reagent was mixed with supernatant in the dark for 15 min according to the Cytotoxicity Detection Kit (Roche, Mannheim, Germany) instructions, and LDH release was measured via a microplate reader (TECAN, Austria) at 492 nm. The cytotoxicity of shionone against A549 cells and J774 cells was determined with the following formula:

LDH release (%) = (OD_sample_
*−* OD_Neg._)/(OD_Pos._
*−* OD_Neg._) × 100%

After determining the cytotoxicity of shionone against mammalian cell lines, the protective effect of shionone against cell injury induced by PLY was also explored. PLY (0.48 μM) was incubated with different concentrations of shionone (2, 4, 8 μg/mL) for 30 min at 37 °C and then cocultured with A549 cells (2 × 10^4^ cells/well) for 6 h. The supernatant was removed after centrifugation at 1000× *g* for 10 min, and dyeing working reagent was added according to the LIVE/DEAD^TM^ Viability/Cytotoxicity Kit (Invitrogen, Carlsbad, CA, USA) instructions in the dark for 40 min. The live cells dyed green and dead cells dyed red were imaged by fluorescence microscopy (Fv1000, Olympus).

### 4.7. PLY Expression Determination

Overnight cultures of *S. pneumoniae* were expanded in THY at 1:100 and incubated to OD_600 nm_ = 0.3. *S. pneumoniae* was cocultured with different concentrations of shionone (2, 4, 8 μg/mL) for 6 h at 37 °C. The bacterial pellet was collected at 10,000× *g* for 2 min and resuspended in 1× SDS-PAGE loading buffer. After heating at 95 °C for 10 min, the samples were separated by 12% SDS-PAGE and transferred to a polyvinylidene fluoride (PVDF) membrane. The PVDF membranes were incubated with the rabbit antipneumolysin monoclonal primary antibody (1:5000; Abcam, San Francisco, CA, USA) at 4 °C for 12 h. After washing with TBST 3 times, the PVDF membranes were incubated with HRP-conjugated AffiniPure goat anti-mouse IgG (H + L) secondary antibodies (1:3000; Proteintech, Rosemount, IL, USA) for 1 h. The PLY expression level of *S. pneumoniae* treated with different concentrations of shionone was imaged after the addition of ECL western blotting substrate (Thermo Scientific™, Rockford, IL, USA).

### 4.8. Oligomerization Analysis

PLY could bind to the cholesterol membranes of cells to causing cytolysis via self- binding oligomers formation [8]. In our study, oligomerization analysis was performed to explore whether shionone could disturb the oligomers formation to neutralize the activiy of PLY. PLY (0.48 μM) was coincubated with different concentrations of shionone (2, 4, 8 μg/mL) in PBS at 37 °C for 1 h. Afterward, 5 × SDS loading buffer without beta-mercaptoethanol was added and incubated for 10 min at 55 °C. Samples were separated by 6% SDS-PAGE and transferred to PVDF membranes. Moreover, PVDF membranes were incubated with the His-Tag Mouse McAb (Proteintech, Rosemont, IL, USA) for 1 h and analyzed with a visualizer after washing 3 times.

### 4.9. Molecular Docking Simulation

The crystal model of PLY protein (4QQA) was taken from the RCSB protein database (https://www.rcsb.org/, accessed on 10, April, 2022). The chemical structure data of shionone were obtained from PubChem (https://pubchem.ncbi.nlm.nih.gov/, accessed on 10 April 2022). The protein receptors were simulated by docking with small molecule ligands by SailVina v1.0 software [26,27]. The results were uploaded to the Protein-Ligand Interaction Profiler (PLIP) website (https://plip-tool.biotec.tu-dresden.de/plip-web/plip/index, accessed on 10 April 2022) for online analysis [28]. Finally, results were visualized using PyMol v2.4.0.

### 4.10. Mouse Pneumonia Model

Six- to eight-week-old female BALB/c mice were purchased from Liaoning Changsheng Biotechnology Co., Ltd. (Changchun, Jilin, China). The study received ethical permission of Jilin University and all animal experimental procedures followed the guidelines of the Animal Care and Use Committee (ACUC) of Jilin University. *S. pneumoniae* was cultured to logarithmic growth phase (OD_600 nm_ = 0.6). Bacterial pellets were collected at 8000× *g* for 10 min at 4 °C and suspended in 3 mL of sterile PBS. The mouse pneumonia model was established by intranasal infection (4 × 10^8^ CFU/mouse). Shionone was given to the treatment group at a dose of 50 mg/kg in 0.5% sodium carboxymethylcellulose solution orally twice a day. After infection for 48 h, the lung tissue was collected for histopathological analysis and colony counting.

### 4.11. Statistical Analysis

All the tests were performed at least 3 times. The experimental data was analyzed by GraphPad Prism 8.0.2 using Student’s *t* tests and were expressed as $\bar{x}$ ± SD (mean ± standard deviation). The significance level was expressed as follows: * *p* < 0.05; ** *p* < 0.01; NS, no significant difference.

## 5. Conclusions

The role of shionone against *S. pneumoniae* infection was explored first. Our results indicated that shionone could neutralize the PLY activity via disturbing the oligomerization and inhibiting the PLY amino acid site ASP-59, ILE-60, THR-57, PHE-344, and ASN-346. At same time, shionone could alleviate pneumonia injury induced by *S. pneumoniae* in vivo. Therefore, shionone is an effective natural compound for the treatment of *S. pneumoniae* infection.

## Data Availability

The data that support the findings of this study are available from the corresponding author upon reasonable request.

## Supplementary Materials

The following supporting information can be downloaded at: https://www.mdpi.com/article/10.3390/molecules27196258/s1, Table S1: The Binding energy data between PLY and shionone.

## Sample Availability

Samples of the compounds that support the findings of this study are available from the corresponding author upon reasonable request.
