# Supplementary material for: Shionone-Targeted Pneumolysin to Ameliorate Acute Lung Injury Induced by Streptococcus pneumoniae In Vivo and In Vitro
Source: Molecules. 2022 Sep 23;27(19):6258. doi: 10.3390/molecules27196258 (PMC9573397; doi:10.3390/molecules27196258)
Supplement: Supplementary file 1 [file molecules-27-06258-s001.zip › molecules-1878800-supplementary.pdf]

Table S1 The Binding energy data between PLY and shionone.

ligand_name	ΔG(kcal/mol)	Ki(nM)
Structure2D_CID_12315507_config37_out1.pdbqt	-12.9	0.342
